# Dual mechanisms regulate ecosystem stability under decade-long warming and hay harvest

**DOI:** 10.1038/ncomms11973

**Published:** 2016-06-15

**Authors:** Zheng Shi, Xia Xu, Lara Souza, Kevin Wilcox, Lifen Jiang, Junyi Liang, Jianyang Xia, Pablo García-Palacios, Yiqi Luo

**Affiliations:** 1 Department of Microbiology and Plant Biology, University of Oklahoma, Norman, Oklahoma 73019, USA; 2 Co-Innovation Center for Sustainable Forestry in Southern China, College of Biology and The Environment, Nanjing Forestry University, Nanjing 210037 China; 3 Oklahoma Biological Survey, University of Oklahoma, Norman, Oklahoma 73019, USA; 4 Tiantong National Forest Ecosystem Observation and Research Station, School of Ecological and Environmental Sciences, East China Normal University, Shanghai 200062, China; 5 Research Center for Global Change and Ecological Forecasting, East China Normal University, Shanghai 200062, China; 6 Área de Biodiversidad y Conservación, Departamento de Biología, Geología, Física y Química Inorgánica, Escuela Superior de Ciencias Experimentales y Tecnología, Universidad Rey Juan Carlos, C/Tulipán s/n, Móstoles 28933, Spain; 7 Center for Earth System Science, Tsinghua University, Beijing 100084 China

## Abstract

Past global change studies have identified changes in species diversity as a major mechanism regulating temporal stability of production, measured as the ratio of the mean to the standard deviation of community biomass. However, the dominant plant functional group can also strongly determine the temporal stability. Here, in a grassland ecosystem subject to 15 years of experimental warming and hay harvest, we reveal that warming increases while hay harvest decreases temporal stability. This corresponds with the biomass of the dominant C_4_ functional group being higher under warming and lower under hay harvest. As a secondary mechanism, biodiversity also explains part of the variation in temporal stability of production. Structural equation modelling further shows that warming and hay harvest regulate temporal stability through influencing both temporal mean and variation of production. Our findings demonstrate the joint roles that dominant plant functional group and biodiversity play in regulating the temporal stability of an ecosystem under global change.

## Abstract (web summary)

Species diversity is thought to play an important role in maintaining production stability. Shi *et al.* demonstrate that the dominant C4 plant also makes a substantial contribution to temporal stability in a grassland ecosystem subject to 15 years of experimental warming and hay harvest.

## Graphical abstract

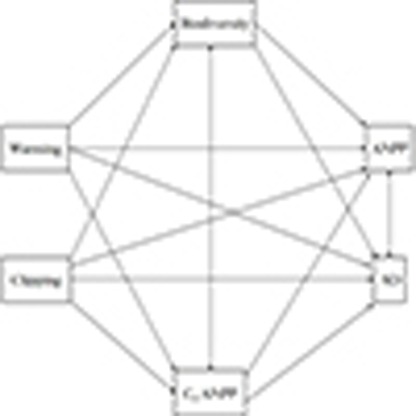

Human activities are driving global biodiversity loss at an unprecedented rate^1^, causing potential declines in ecological functioning and associated ecosystem services. Theory predicts that the temporal stability of ecosystem production increases with biodiversity through a variety of mechanisms including overyielding, species asynchrony and portfolio effects^2^,^3^. Further, a recent synthesis of 12 manipulative experiments showed the impacts of global change (that is, changes in plant diversity, nitrogen, carbon dioxide, fire, herbivory and water) on temporal stability of production through changes in species diversity^4^. Additional to biodiversity, the abundance of dominant plant functional groups can be altered under global change scenarios and has been proposed to regulate ecosystem function^5^,^6^ and temporal stability^5^,^7^,^8^. However, to date, there is little experimental evidence in global change experiments to support the role of dominant plant functional group in regulating the temporal stability of production.

Results from global change experiments^9^,^10^, long-term isotope data^11^ and large-scale model predictions^12^,^13^ suggest that warming shifts functional group composition by strengthening C_4_ species dominance over C_3_ species in grassland ecosystems. Due to the inherent high water and nutrient use efficiency^6^,^10^,^14^, C_4_ species are expected to be more stable than C_3_ species, especially in a highly variable climate. Therefore, global warming could stabilize the function of future grassland ecosystems through increased C_4_ abundance. However, a shift in functional group composition under warming is often accompanied by other changes in plant community structure, which may compound or attenuate the effects of functional group shifts on ecosystem stability. For instance, experimental warming was reported to induce species loss^15^,^16^, which is likely to decrease ecosystem production and temporal stability^4^,^17^. Similar to warming, land management, such as hay harvest in Great Plains grasslands, can alter multiple attributes of plant communities^18^ and further affect ecosystem stability. For example, mowing or grazing often increases species richness by increasing light availability^19^,^20^,^21^ and suppressing growth of dominant species^22^. Plant communities with higher diversity are likely to be more productive and temporally stable^23^,^24^. It is thus critical to understand how land management will mediate future climate's impact on grassland production and temporal stability.

Our ongoing, long-term, warming and clipping (mimicking hay harvest) experiment in a grassland ecosystem has addressed ecosystem-level responses of ecosystem C fluxes^25^,^26^ and stocks^10^,^27^, as well as community-level responses of plant composition^18^. Here, we analysed long-term temporal stability of aboveground net primary production (ANPP) in response to warming and clipping over a 15-year period (2000–2014) and associated mechanisms. Annual precipitation within the study period covered the driest (515 mm) and wettest (1307, mm) year in the last 55 years, with an average of 874 mm (Supplementary Figs 1a and 2). This strong variability in background climate provided us with a unique opportunity to investigate the temporal stability of production under warming and clipping emulating hay harvest. Annual mean air temperature spanned ca. 3 °C difference from 15.1 to 17.7 °C, with a mean of 16.1 °C. Across the 15 years, experimental warming elevated soil temperature by 1.3 °C on average in the unclipped plots and 2.2 °C on average in the clipped plots (Supplementary Fig. 1b). Clipping increased soil temperature by 0.5 and 1.3 °C in control and warmed plots, respectively. Warming decreased soil water content by 1.5% on average across the 15 years and clipping decreased soil water content by 0.6% on average (Supplementary Fig. 1c). Warming did not interact with clipping to impact soil water content (Supplementary Table 1).

Previous studies in this experimental site reported that warming enhanced ANPP, while clipping suppressed ANPP mainly through impacts on C_4_ plant functional group production^10^,^27^. We first hypothesized that temporal stability of C_4_ production would be greater than that of C_3_ production, regardless of experimental treatments. Accordingly, we further hypothesized that due to their opposing impacts on C_4_ plant production, warming would enhance the temporal stability of ANPP, while clipping would decrease the temporal stability.

## Results

### Treatment effects on ANPP and its temporal stability

**Figure 1:**
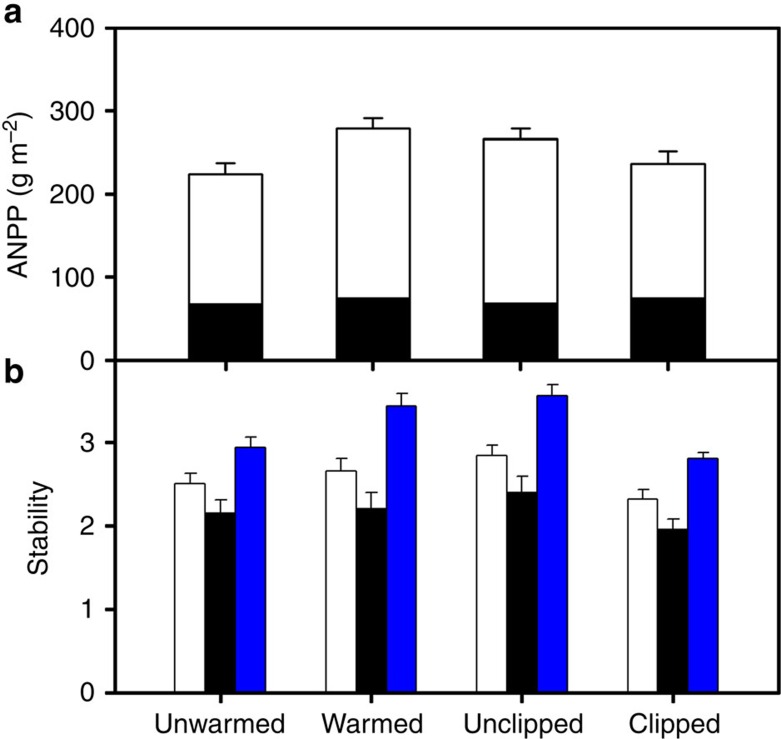
Treatment effects on ANPP and its temporal stability. Effects of warming and clipping on (**a**) mean annual C_4_ ANPP (white bar), C_3_ ANPP (black bar) and community ANPP, and (**b**) temporal stability of C_4_ ANPP (white bar), C_3_ ANPP (black bar) and community ANPP (blue bar) within 2000–2014. Warming and clipping had opposing effects on ANPP through affecting C_4_ ANPP (**a**). Temporal stability of C_4_ ANPP was consistently greater than that of C_3_ ANPP across all the treatments (*n*=12; unwarmed: *t*=−1.86, *P*=0.08; warmed: *t*=−2.74, *P*=0.02; unclipped: *t*=−2.60, *P*=0.02; clipped: *t*=−1.88, *P*=0.07); warming therefore increased but clipping decreased the temporal stability of ANPP (**b**). Error bars represent s.e. across replicates (*n*=12).

During the 15-year period, ANPP showed strong interannual variability with a threefold difference among years (Supplementary Fig. 3). First of all, warming and clipping did not interact to affect total ANPP, C_3_ or C_4_ ANPP (Supplementary Table 1) and their temporal stability (Table 1). Warming was a strong stabilizing force and enhanced ANPP and its temporal stability through promoting C_4_ plant production. ANPP and C_4_ ANPP were 24.9% and 30.6% greater in the warmed plots than in the unwarmed plots, respectively, over the 15 years (Fig. 1a). In contrast, ANPP and C_4_ ANPP were 11.5% and 18.3% lower in the clipped plots than in the unclipped plots, respectively (Fig. 1a). Contrary to our treatment effects on C_4_ ANPP, neither warming nor clipping had significant impacts on C_3_ ANPP (Fig. 1a). Our findings are consistent with model predictions^12^,^13^ and other climate change experiments from a desert steppe^28^ and a mixed-grass prairie^9^,^29^, where C_4_ plant production responded positively to experimental warming and C_3_ production remained unresponsive. Clipping has also been found to decrease ecosystem production by inhibiting dominant C_4_ grasses^22^.

As expected, the temporal stability in C_4_ ANPP was greater than C_3_ ANPP (paired *t*-test; df=23; *t*=−3.26; *P=*0.003) on average 18.3% across all treatments with marginal significance (paired *t*-test; *P*<0.1) in the unwarmed (paired *t*-test; *P=*0.08) and clipped treatments (paired *t*-test; *P=*0.07) (Fig. 1b), primarily because C_4_ species had greater production due possibly to the inherent higher water and nitrogen use efficiency^6^,^10^,^14^. Due to opposing effects of warming and clipping on C_4_ ANPP, warming enhanced the temporal stability of ANPP by 17.8%, whereas clipping decreased it by 20.4% (Table 1; Fig. 1b). The temporal stability of C_4_ and C_3_ ANPP did not differ between warmed and unwarmed plots (Table 1; Fig. 1b); however, clipping reduced the stability of C_4_ ANPP possibly because clipping increased species richness but reduced community evenness (Supplementary Table 1) with increase in relative abundance of one dominant species and decrease in relative abundance of two subdominant species.

### Relationships between plant community and temporal stability

**Figure 2:**
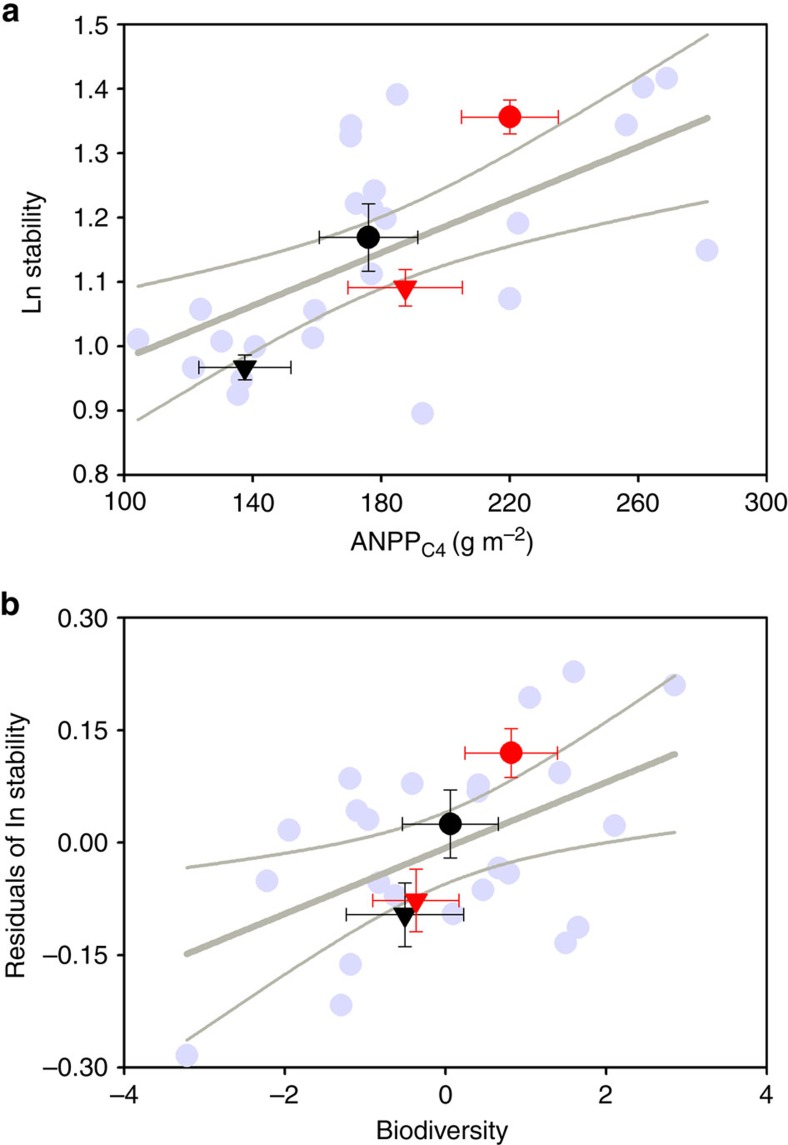
Relationships between plant community and the temporal stability of ANPP. The relationship between mean annual C_4_ ANPP (**a**) and biodiversity (**b**), and the temporal stability of ANPP within 2000–2014. The temporal stability is positively dependent on mean annual C_4_ ANPP (*F*_1, 22_=13.7, *P*=0.001) and the residuals of the stability after controlling for C_4_ ANPP effect is positively associated with biodiversity (*F*_1, 22_=7.83, *P*=0.01). Both factors together explained 56% variations in the temporal stability. Grey circles represent values in each of the experimental plots. The black symbols are the mean values with s.e.'s across replicates (*n*=6) for plots under ambient temperature and the red symbols are the mean values with s.e.'s across replicates (*n*=6) from warmed plots; the triangles are the mean values with s.e.'s across replicates (*n*=6) from clipped plots and the black and red circles are the mean values with s.e.'s across replicates (*n*=6) from unclipped plots. Grey line shows linear fit with 95% confidence interval. Here the biodiversity was the first component of a principal component analysis with richness, evenness and diversity (Supplementary Fig. 4).

Multiple linear regression (MLR) analysis revealed positive association between temporal stability of ANPP and mean annual C_4_ production (Table 2; Fig. 2a). This finding underlines the importance of plant functional group in addition to biodiversity in determining the temporal stability of production. These results are in line with field experiments, paleoecological and isotopic evidence supporting that C_4_ species are considered well adapted to a warmed climatic condition^11^,^30^,^31^. Besides C_4_ production, biodiversity as a secondary mechanism also significantly explained part of the variation in the temporal stability of ANPP (Table 2; Fig. 2b). Here the biodiversity variable is the first component (PC1) of a principal component analysis (PCA) with species richness, evenness and Shannon–Wiener diversity. This biodiversity mechanism was more contributed by community evenness and diversity, and less by species richness (Supplementary Fig. 4). Overall, C_4_ ANPP explained 38% of the variation in temporal stability, and PC1 (the combination of increased Shannon–Wiener diversity and evenness and lower richness) explained an additional 18%, suggesting that, across the warming and clipping treatments, the mean abundance of C_4_ was the strongest predictor. Positive relationships between species richness and ecosystem function and stability are often reported in studies, yet many of these studies were conducted in assembled grasslands with direct manipulation in species richness^32^. In natural grasslands, however, different forms of diversity–function–stability relationships are typically reported^33^, indicating the greater complexity of real-world ecosystems (but see ref. 23 for positive relationship between species richness and the temporal stability in natural grasslands).

### Direct and indirect treatment effects on temporal stability

**Figure 3:**
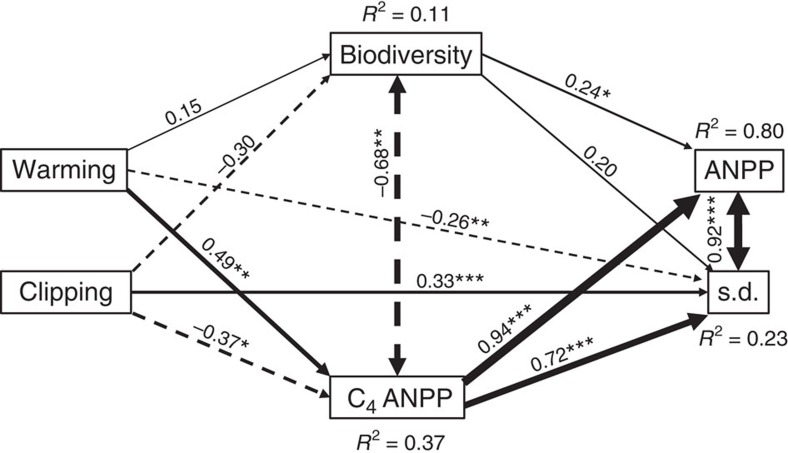
Direct and indirect treatment effects on the two stability components. Structural equation model depicting the direct and indirect effects of warming, clipping, biodiversity and C_4_ ANPP on the two components of the temporal stability (temporal mean of ANPP and temporal s.d. of ANPP). Boxes indicate measured variables entered in the model. Numbers adjacent to arrows are standardized path coefficients, analogous to partial regression weights and indicative of the effect size of the relationship. Continuous and dashed arrows indicate positive and negative relationships, respectively. Single-headed arrows represent causal relationships, and double-headed arrows represent covarying variables. The path widths are scaled proportionally to the path coefficients. As in other linear models, *R*^2^ indicates the proportion of variance explained. The SEM used satisfactorily fitted our data, as suggested by results of goodness-of-fit tests (*χ*^2^=3.554, *P*=0.314; NFI=0.971; RMSEA=0.090, *P*=0.339; ref. 37). To increase the degrees of freedom, paths from warming and clipping to ANPP were removed from the model, as their path coefficients were very small (<0.10) and not significant (*P*>0.05). ***P<0.001; **P<0.01; *P<0.05. S.d. was natural-logged to increase linearity with predictors. Here the biodiversity was the first component of a principal component analysis with richness, evenness and diversity (Supplementary Fig. 4).

The structural equation modelling (SEM) showed that warming and clipping had no direct effects on mean annual ANPP (SEM; path coefficients <0.10 and *P*>0.05), but they had indirect effects via C_4_ ANPP and biodiversity (Table 3; Fig. 3). Warming increased C_4_ ANPP and biodiversity (SEM; path coefficient >0.1, but *P*>0.05), which in turn increased mean annual ANPP (Fig. 3). Thus, the total effects of warming on mean annual ANPP were positive (0.50). Clipping reduced C_4_ ANPP and biodiversity (SEM; path coefficient >0.1, but *P*>0.05), which in turn increased mean annual ANPP. Thus, the total effects of clipping on mean annual ANPP were negative (−0.42). Warming reduced, whereas clipping increased standard deviation (s.d.) of ANPP via direct effects (Fig. 3). Interestingly, indirect treatment effects on s.d. via changes in C_4_ ANPP and biodiversity counteracted such direct effects (Table 3). As C_4_ ANPP and biodiversity increased s.d., the negative effects of warming were cancelled, and the total effects were positive (0.12). The negative relationship between clipping and plant community reduced the total effects on s.d. to almost neutral (0.01).

The total treatment effects on mean annual ANPP, positive for warming and negative for clipping, were larger in magnitude than the total effects on s.d., due to the counteracting role of indirect effects mediated by plant community (Table 3). Thus, warming and clipping mainly affected the temporal stability of ANPP (ratio of temporal mean ANPP to s.d.) via changes in mean annual ANPP and not in s.d. When evaluated together, these results suggest that the overall effects of warming on the temporal stability of ANPP is positive, whereas the overall effect of clipping is negative, supporting our results from Fig. 1b. The SEM also confirmed the role of C_4_ ANPP modulating warming and clipping effects on the temporal stability of ANPP. On the other hand, biodiversity emerged as a secondary mechanism modulating treatment effects, especially clipping (Table 3). Our results showed novel dual mechanisms regulating the temporal stability of ANPP under decade-long warming and hay harvest, but future research is needed to explicitly test our findings by larger experiments jointly manipulating global change drivers and plant community attributes.

The regulatory role of biodiversity change by global change drivers on temporal stability has been reported in several grassland ecosystems^4^,^29^. In particular, our finding is highly comparable to a long-term global change study in which elevated CO_2_ enhanced the temporal stability through increasing the evenness and diversity of the plant community by suppressing dominant species in a mix-grass prairie^29^. Furthermore, direct manipulation of community evenness and diversity also demonstrated that higher biodiversity lead to greater temporal stability^34^,^35^. The role of both plant functional group and biodiversity in determining the effects of warming and land management on ecosystem temporal stability again highlights the complex nature of stability, the controls of which are the basis of a long-standing debate in ecology.

## Discussion

Our study reported, for the first time, dual mechanisms regulating the temporal stability of production under global change. Climate- and land management-induced changes in dominant plant functional group deserve great attention alongside with biodiversity when managing communities for the maintenance and temporal stability of ecosystem function. In particular, C_4_ species have been predicted to dominate many grassland ecosystems at the expense of C_3_ species in future climatic scenarios^12^,^13^. Therefore, grassland ecosystem stability may be strengthened in the future, providing greater forage or feedstock provision and predictability of important ecosystem function such as carbon cycling or sequestration. However, alterations in biodiversity should also be considered as it may mediate the strength of temporal stability responding to global change. Anthropogenic perturbations, especially those that can adversely affect biodiversity, may attenuate and even override increases in temporal stability caused by dominant plant functional group change. In addition, climate change will likely be multi-faceted, and these simultaneous changes have the potential to interact, causing novel states of ecosystem functioning and stability. Opposite to warming effect, management practice of haying emulated by clipping in this study had negative impact on dominant functional group abundance and biodiversity and led to low temporal stability of production, which is likely to cause deterioration in ecosystem services provided by grasslands. Overall, these findings demonstrate the roles of different attributes of a plant community in regulating temporal stability of an ecosystem in the face of various global changes.

## Methods

### Study site and design

The warming and clipping (mimicking hay harvest) experiment has been conducted on the Kessler Atmospheric and Ecological Field Station (KAEFS) of the University of Oklahoma (34° 58′ 54′′ N and 97° 31′ 14′′ W) in the Southern Great Plains of USA. The grassland was dominated by the C_4_ grasses (*Schizachyrium scoparium* and *Sorghastrum nutans*) and the C_3_ forbs (*Ambrosia psilostachya*, *Solidago nemoralis* and *Solidago rigida*). The experiment has four treatments: unclipping and control (ambient) temperature (UC), unclipping and warming (UW), clipping and control temperature (CC), and clipping and warming (CW) with warming as the main factor and clipping nested within warming treatment in a paired factorial split-plot design. Six pairs of plots of 2 × 2 m were subjected to continuous warming using infrared heaters versus the control with ambient temperature since November 1999. Each 2 × 2-m plot is divided into four 1 × 1-m subplots. Plants in two diagonal subplots are clipped at a height of 10 cm above the ground once a year in August to mimic hay harvest, while the other two subplots are unclipped. For more details, see ref. 27.

### Aboveground primary production

The ANPP was estimated once a year at peak biomass, usually in August. We chose this time to obtain the most accurate estimates of production. ANPP was estimated using the pin-contact method. A 0.5-m tall frame holding 10 pins at a 60° angle was placed in each subplot four times (once in each of the four cardinal directions). Pins could be pulled up to give data up to 1 m above the soil surface. Every time a piece of plant material hit a pin it was counted as one hit of either C_3_ or C_4_ species. The contact numbers were then used to estimate aboveground biomass using calibration equations derived from 10 calibration plots for C_3_ and C_4_ species, which were randomly selected each season and year and located at least 5 m away from the experimental plots. Biomass in the calibration plots was clipped to the ground surface. Clipped plant material was oven-dried and then correlated with the total contact number for C_3_ and C_4_ species. A linear regression between contact number and biomass was used to derive the calibration equation. *R*^2^ of these biomass-contact equations varied from 0.5 to 0.95 depending on treatment and year and averaged 0.72 over all treatments. These equations were then used to estimate the biomass of C_3_ and C_4_ species in the experimental subplots. More details are described in ref. 27.

### Community structure and temporal stability of ANPP

Species richness (*S*) was calculated as the total number of plant species in the two subplots. Relative abundance of each species is calculated as the ratio between hits for the given species and total hits for all the species in the two subplots. We calculated the Shannon–Wiener diversity index (H′) as 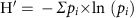, where *p*_*i*_ is the relative abundance of species *i*, and evenness (*E*) as 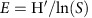.

Temporal stability of total ANPP, C_4_ and C_3_ were calculated as the ratio between mean annual ANPP and its s.d. over time.

### Statistical analysis

We performed a two-way analysis of variance (ANOVA) to examine the main and interactive effects on temporal stability of ANPP (total, C_3_ and C_4_), with warming (main plot) and clipping (subplot) as main effects, and plot pair as a random effect. Temporal stability was natural logged to ensure normality and homogeneity. We used repeated-measures ANOVA to examine the main and interactive effects on soil temperature, soil water content, species richness, diversity, and evenness and ANPP (total, C_3_ and C_4_), with warming (main plot) and clipping (subplot) as main effects, year as the repeated factor and plot pair as a random effect. A paired *t*-test was applied to compare the temporal stability of production between C_3_ and C_4_ plant functional group under each treatment.

We used stepwise MLR in PROC REG of SAS for model selection to investigate the effects of abiotic (soil temperature and soil water content) and biotic (C_4_ ANPP, species richness, evenness and Shannon–Wiener diversity) factors on the temporal stability of ANPP. We specified *α=*0.05 as the significance cutoff for the variables to enter the regression model and *α*=0.1 as the cutoff for the variables to stay in the model. To avoid redundancy among highly correlated variables, we conducted a PCA with species richness, evenness and Shannon–Wiener diversity. Only the first component of the PCA (hereafter called ‘biodiversity') was retained as the biodiversity proxy for the stepwise MLR because it has an eigenvalue (2.25) >1 and accounted for 75% variation in the data (Supplementary Fig. 4). Evenness contributed the largest loading (0.66) to the biodiversity proxy, followed by diversity (0.59) and species richness (−0.46). All statistical analyses were conducted in SAS v.8.1 (SAS Institute Inc., Cary, NC, USA).

To investigate whether warming and clipping affect the temporal stability of ANPP through changes in C_4_ ANPP and biodiversity, we used structural equation modelling (SEM). Following current concepts of the temporal stability, and results from ANOVA and MLR, we proposed an *a priori* model of hypothesized relationships within a path diagram (Supplementary Fig. 5), allowing a causal interpretation of the model outputs^36^. Specifically, we constructed *a priori* conceptual structural equation model depicting the direct and indirect effects of warming, clipping, plant community diversity and C_4_ ANPP on the two components of the temporal stability of ANPP (temporal mean ANPP and temporal s.d. of ANPP) (Supplementary Fig. 5). ‘Warming' and ‘clipping' were represented as binary variables coding for control temperature and warming levels, and unclipping and clipping levels, respectively. Categorical exogenous variables are compatible with structural equation models because distributional assumptions do not apply to them^36^. To increase the degrees of freedom, any path with a coefficient <0.10 was removed from the model when not significant (for example, ‘warming' to ‘C_4_ ANPP' and ‘clipping' to ‘C_4_ ANPP'). Path coefficients were obtained using the maximum likelihood estimation technique. S.d. was natural log-transformed to increase linearity with predictors. We used the traditional *χ*^2^ goodness-of-fit test, but because of its sensitivity to sample size, the NFI and RMSEA indices were also considered^36^ and checked according to ref. 37.

We focused on the two components of the temporal stability of ANPP (temporal mean and s.d. of ANPP) as our two response variables, to disentangle whether warming and clipping influenced temporal stability via changes in temporal mean and/or temporal variation. Considering our low sample size (24 plots), we excluded soil temperature and soil water content from the model, as they were not selected by the stepwise MLR. The first component of the community structure PCA was used as the biodiversity proxy. As the total treatment effects are the sum of direct plus indirect effects^36^, the percentage of the total effects indirectly modulated by C_4_ ANPP and biodiversity can be calculated. We also calculated the percentage of the treatment indirect effects modulated by biodiversity and C_4_ ANPP separately. The PCA was performed using JMP 10.0 (SAS Institute Inc.) and the SEM analyses were performed with AMOS 22.0 (Amos Development Co., Armonk, NY, USA).

### Data availability

All data are available from  http://ecolab.ou.edu/download/Shi_MS_Under_Review.php

## Additional information

**How to cite this article:** Shi, Z. *et al.* Dual mechanisms regulate ecosystem stability under decade-long warming and hay harvest. *Nat. Commun.* 7:11973 doi: 10.1038/ncomms11973 (2016).

## Supplementary Material

Supplementary Information Supplementary Figures 1-5, Supplementary Table 1

## Figures and Tables

**Table 1:** Results of two-way ANOVA (*F* and *P* values) for responses of temporal stability of community, C_4_,and C_3_ ANPP to warming, clipping and their interactions.

	**df**	**Stability ANPP**		**Stability C**_**4**_ **ANPP**		**Stability C**_**3**_ **ANPP**	
		***F***	***P***	***F***	***P***	***F***	***P***
W	1, 5	20.79	**0.006**	0.64	0.46	0.05	0.82
C	1, 10	79.10	**<0.0001**	28.26	**0.0003**	4.21	0.07
W × C	1, 10	3.06	0.12	1.28	0.28	1.28	0.28

ANPP, aboveground net primary production; ANOVA, analysis of variance.

Significant results (*P*<0.05) are bolded.

**Table 2:** Results of stepwise multiple linear regression for the effects of abiotic factors (soil temperature (ST) and soil water content (SWC)) and biotic factors (C_4_ ANPP and biodiversity) on the temporal stability of ANPP.

	**Estimates**	***t*** **value**	***P***-**value**	**Partial** ***R***^**2**^
C_4_ ANPP	0.003	4.988	0.000	0.38
Biodiversity	0.049	2.910	0.008	0.18
Intercept	0.689	7.280	0.000	—

ANPP, aboveground net primary production.

ST and SWC were excluded by the stepwise multiple linear regression. Here the biodiversity was the first component of a principal component analysis with richness, evenness and diversity (Supplementary Fig. 4).

df=2, 21; *F* value=13.38; *P*<0.001; *R*^2^=0.56.

**Table 3:** Standardized total, direct, and indirect effects of warming and clipping on the two components of the temporal stability (temporal mean of ANPP and temporal s.d. of ANPP), as derived from the structural equation model of Fig. 3.

**Stability components**	**Treatment**	**Total effect**	**Direct effect**	**Indirect effect**	**% Indirect effect modulated by C**_**4**_ **ANPP**	**% Indirect effect modulated by biodiversity**
ANPP	Warming	0.50	0	0.50	92.8	7.2
	Clipping	−0.42	0	−0.42	82.8	17.2
s.d.	Warming	0.12	−0.26	0.38	92.2	7.8
	Clipping	0.01	0.33	−0.32	81.6	18.4

ANPP, aboveground net primary production.

The percentage of the treatment indirect effects modulated by biodiversity and C_4_ ANPP separately, are also shown. All paths with coefficients<0.10 and *P*>0.05 (for example, warming and clipping→ANPP) were removed from the model to increase the degrees of freedom, and thus treatments only affected ANPP via indirect effects. Here the biodiversity was the first component of a principal component analysis with richness, evenness and diversity (Supplementary Fig. 4).
