# Supplementary material for: Dual mechanisms regulate ecosystem stability under decade-long warming and hay harvest
Source: Nat Commun. 2016 Jun 15;7:11973. doi: 10.1038/ncomms11973 (PMC4912621; doi:10.1038/ncomms11973)
Supplement: Supplementary Information — Supplementary Figures 1-5, Supplementary Table 1 [file ncomms11973-s1.pdf]

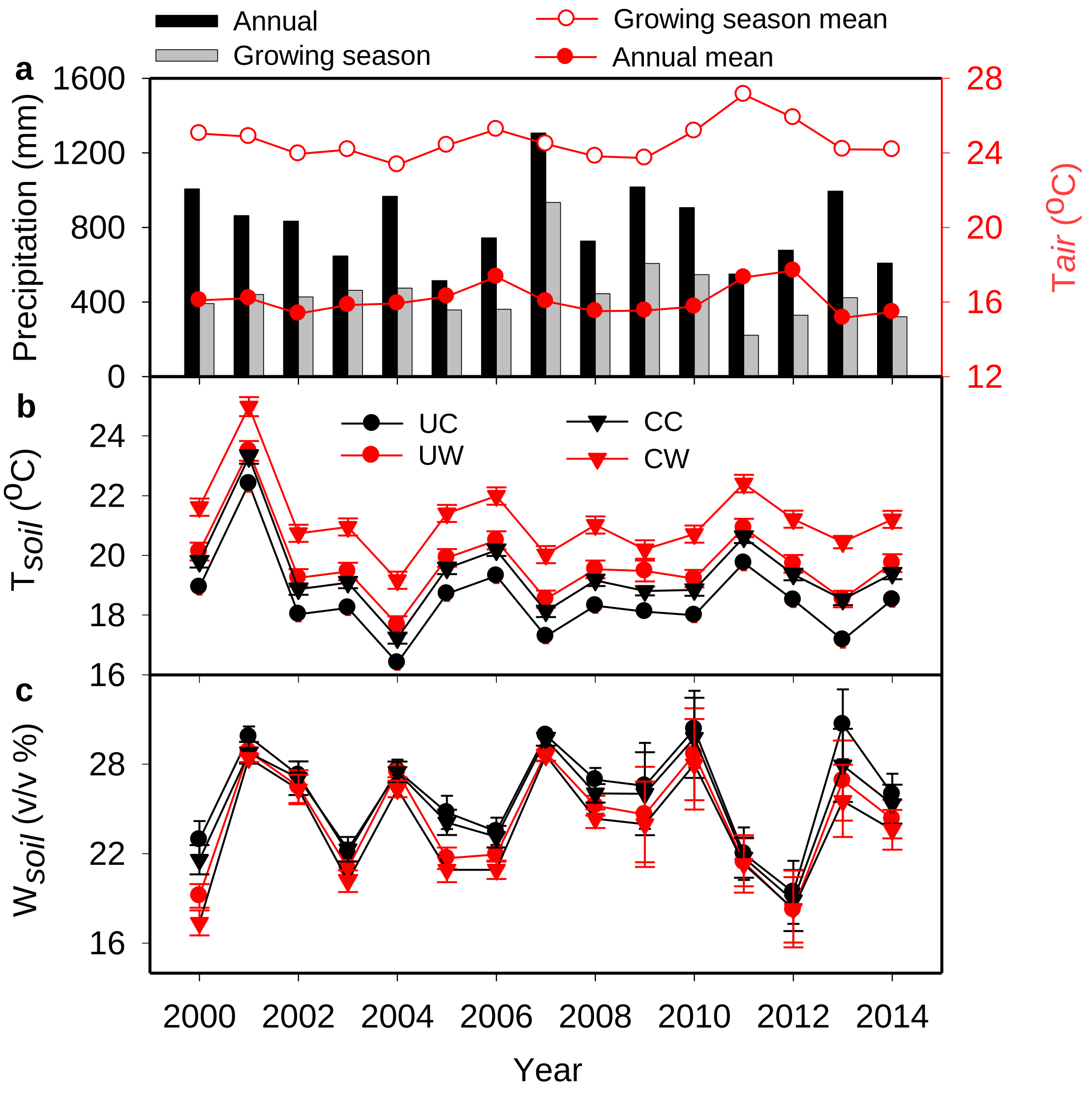

Supplementary Figure 1 Precipitation and air temperature from 2000 - 2014 (a), effects of warming and clipping on soil temperature (b) and soil water content (c) from 2000 - 2014. Treatments are: UC, unclipped with control (ambient) temperature; UW, unclipped and warmed; CC, clipped with control temperature; CW, clipped and warmed. See Supplementary Table 1 for statistics of treatment effects.

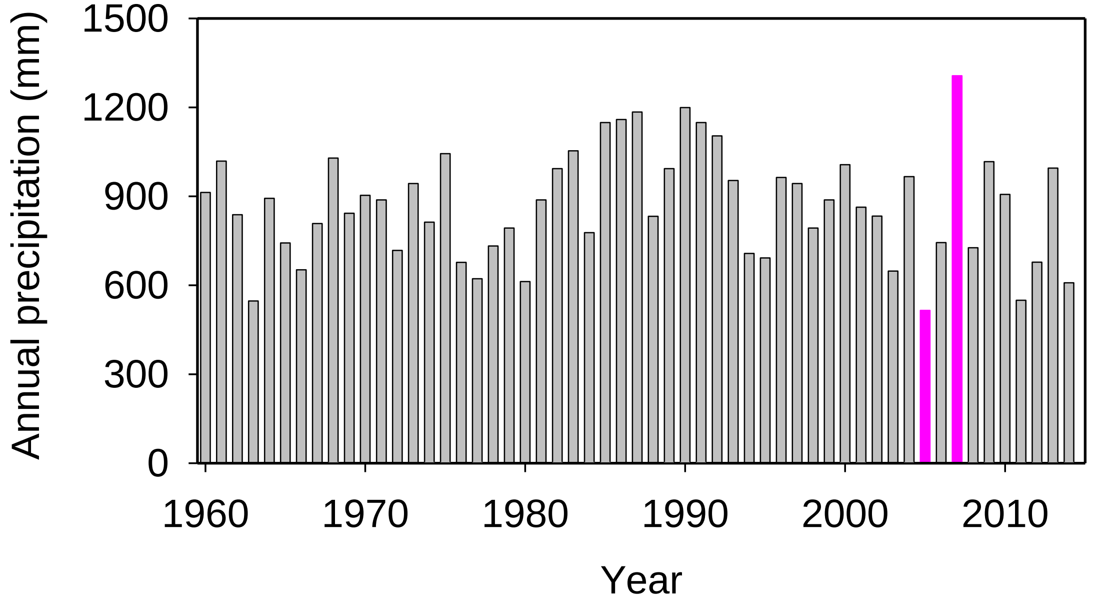

Supplementary Figure 2 Annual precipitation from 1960 to 2014. The two pink bars are from year 2005 and 2007. The 55 years of precipitation data was obtained from the Oklahoma Climate Survey (http://climate.ok.gov/cgi-bin/public/climate.timeseries.one.cgi) from 1960 to 1993 and an Oklahoma Mesonet Station (Washington Station, 200 m away from the study site) from 1994 to 2014.

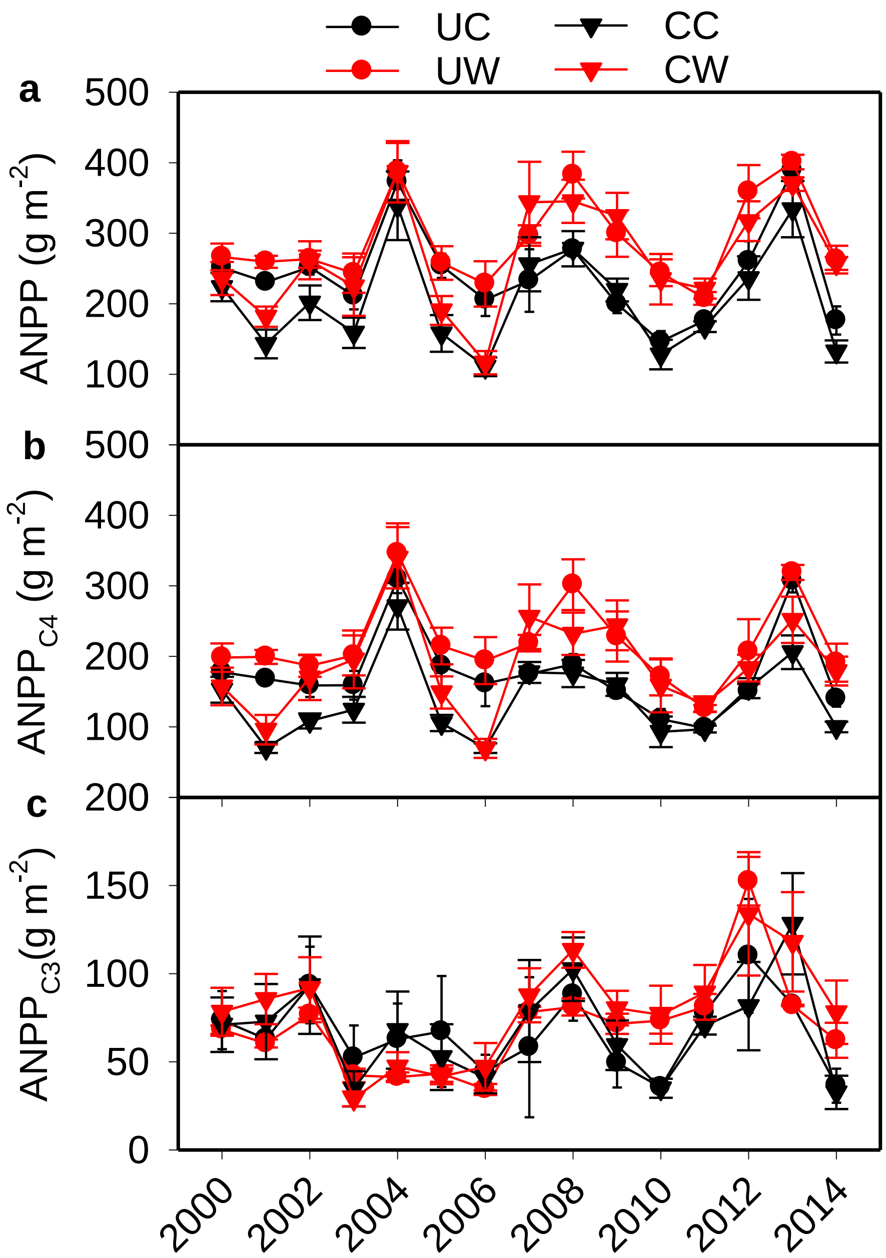

Supplementary Figure 3 Effects of warming and clipping on total ANPP (a), C_4 ANPP (b) and C_3 ANPP (c) from 2000 - 2014. See Supplementary Fig. 1 for treatment abbreviations and Supplementary Table 1 for statistics of treatment effects.

.

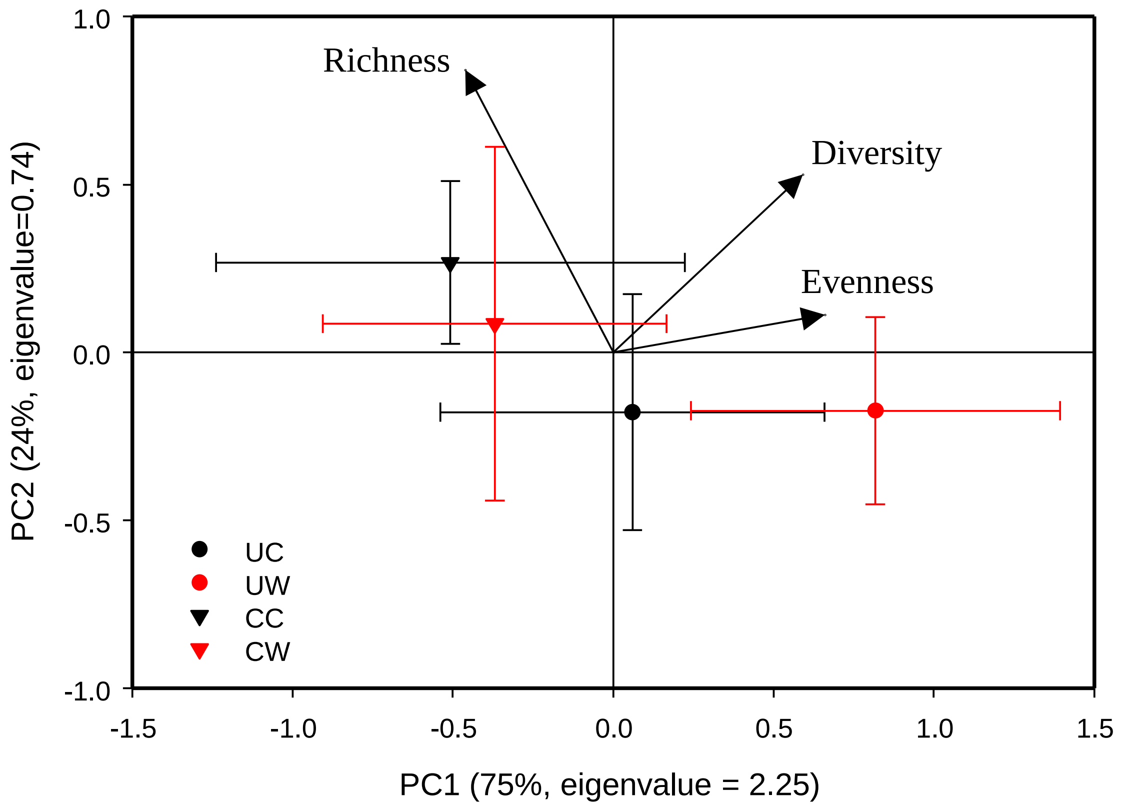

Supplementary Figure 4 Principal component analysis of plant species richness, evenness and diversity (Shannon-Wiener index). Variance explained by each principal component (PC) and its respective eigenvalue is shown in brackets. Values represent means ± SE (n = 6) under the four treatments. Arrows depict the loads of Pearson correlation between richness, evenness and diversity, and the two PC. Only the PC1 was used in both multiple linear regression and structural equation modeling as it has an eigenvalue larger than 1 and explained most of the variance in richness, evenness and diversity.

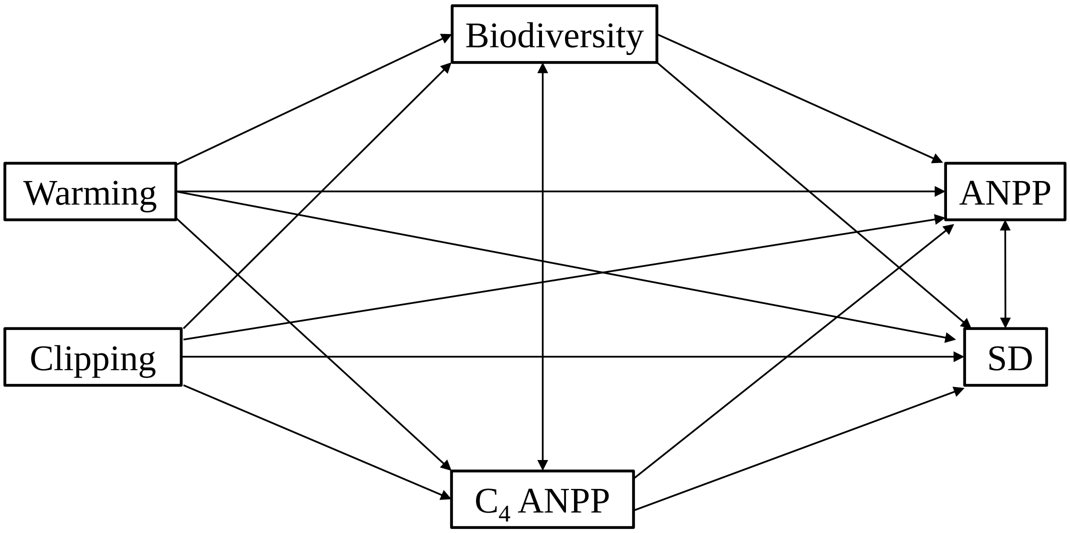

Supplementary Figure 5 A *priori* conceptual structural equation model depicting the direct and indirect effects of warming, clipping, plant community diversity and C_4 ANPP on the two components of the temporal stability of ANPP (temporal mean ANPP and temporal standard deviation (SD) of ANPP). Boxes indicate measured variables entered in the model. Single-headed arrows represent causal relationships, and double-headed arrows represent covarying variables.

Supplementary Table 1 Results of repeated-measures ANOVA (*F* and *P* values) for responses of soil temperature (T_{soil}), soil water content (W_{soil}), species richness (S), Shannon-Wiener diversity index (H'), evenness (E), ANPP and its functional proportion to warming (W), clipping (C), year (Y), and their interactions; Significant results (P < 0.05) are bolded.

	df	**T_{soil}**		**W_{soil}**		**S**		**H'**		**E**		**ANPP**		**C_4 ANPP**		**C_3 ANPP**	
		F	*P*	*F*	*P*	*F*	*P*	*F*	*P*	*F*	*P*	*F*	*P*	*F*	*P*	*F*	*P*
W	1, 5	131.80	**<0.0001**	42.00	**0.001**	1.20	0.33	0.30	0.60	0.40	0.56	30.00	**0.003**	5.81	**0.04**	0.30	0.61
C	1, 290	119.10	**<0.0001**	56.70	**<0.0001**	16.00	**<0.0001**	0.08	0.77	17.20	**<0.0001**	12.50	**0.005**	26.50	**<0.0001**	2.06	0.15
W x C	1, 290	7.60	**0.0063**	0.07	0.80	0.04	0.85	0.59	0.48	1.45	0.28	0.63	0.43	0.18	0.67	0.99	0.32
Y	14, 290	2100	**<0.0001**	35.20	**<0.0001**	35.0	**<0.0001**	11.80	**<0.0001**	18.1	**<0.0001**	58.10	**<0.0001**	49.37	**<0.0001**	17.83	**<0.0001**
W x Y	14, 290	1.40	0.13	0.79	0.66	1.77	**0.05**	0.30	1.00	0.46	0.94	4.16	**<0.0001**	1.87	**0.03**	3.52	**<0.0001**
C x Y	14, 290	9.10	**<0.0001**	0.20	1.00	0.77	0.75	1.98	**0.02**	1.36	0.17	4.83	**<0.0001**	6.14	**<0.0001**	1.97	**0.02**
W x C x Y	14, 290	1.90	**0.03**	0.08	1.00	0.08	1.00	1.52	0.11	1.33	0.18	0.50	0.93	0.80	0.67	0.20	1.00
